# Binding of Tetrachloroaurate(III) to Bovine or Human γ-Globulins

**DOI:** 10.3390/ijms27010541

**Published:** 2026-01-05

**Authors:** Daniil N. Yarullin, Olga I. Logacheva, Maksim N. Zavalishin, George A. Gamov

**Affiliations:** Department of General Chemical Technology, Ivanovo State University of Chemistry and Technology, Sheremetevskii Ave. 7, 153000 Ivanovo, Russia; yarullin_dn@isuct.ru (D.N.Y.); o.logachiova@yandex.ru (O.I.L.); zavalishin00@gmail.com (M.N.Z.)

**Keywords:** serum γ-globulin, gold(III), association equilibrium constant

## Abstract

The interaction of metals with serum γ-globulins is of particular interest, as it can modulate immune system function and lead to unforeseen consequences following the intake of metal ions or their complexes, which are often considered (pro)drugs. This paper focuses on the interactions between gold(III) species and bovine or human serum γ-globulins in aqueous solutions. Using UV-Vis, fluorescence, and CD (circular dichroism) spectroscopy in diluted or 0.1 M NaCl aqueous solutions, we determined the most probable stoichiometry of the gold(III)-protein associates and their conditional binding constants. On average, 13 to 19 gold atoms bind per protein molecule, depending on the medium and protein origin, with apparent binding constants ranging from 3.6 to 4.6 (log K values; hydroxyl-containing complexes exhibit lower binding affinity). CD spectra revealed no changes in protein secondary structure induced by the increase in electrolyte concentration. However, the addition of gold(III) species resulted in a decrease in β-sheet content and a corresponding increase in turns or disordered fragments.

## 1. Introduction

In mammals, globulins comprise both carrier proteins and immunoglobulins (antibodies). The γ-globulin fraction is the heaviest among blood serum proteins separated by electrophoresis [1]. Its primary physiological role is antibody activity, a crucial defensive mechanism in animals [2]. Consequently, chemical interactions between γ-globulins and various xenobiotics, such as drugs and metal ions, can adversely affect the organism’s immune response. In particular, metal binding to antibodies can significantly alter their stability. This is especially true for redox-active ions like Cu^2+^ [3,4], where ROS generated via Fenton-like reactions can induce hinge fragmentation. The conformational state of antibodies, which is directly linked to their biological function, is highly susceptible to the presence of metal ion. It could be observed even with atomic force microscopy [5]. Furthermore, metal-induced protein aggregation represents another critical factor that may impair γ-globulin activity [6].

Among metals suitable for medicinal use and likely to enter the organism, gold attracts significant interest. This is due to the many promising antitumor agents synthesized from gold(III) complexes [7,8,9,10,11], which are isostructural and isoelectronic to the established anticancer drug cisplatin. The antibacterial potential of gold(III)-based organometallic compounds is also under active investigation [12,13,14,15], and gold coatings are already utilized in surgical instruments [16]. Catalytic properties of gold(III) (manifesting in, e.g., [3+2] cycloaddition reactions [17]) may also be of biological relevance. It is important to note, however, that gold(III) complexes typically function as prodrugs rather than active pharmaceutical agents; they require prior activation to exert a biological effect [18]. This activation may involve ligand substitution in the gold coordination sphere (e.g., via hydrolysis) or redox processes (oxidation/reduction). As our previous work has demonstrated [19,20], the dissociation of a metal complex in the presence of a protein can lead to the formation of a metal-protein associate. Therefore, equilibria involving metal ions (or their simple complexes, such as tetrachloroaurate(III), since Au^3+^ itself cannot persist in aqueous solution [21]) with proteins must be taken into account.

Data on the interaction of metal ions with γ-globulins are extremely scarce. In an early study, Bondareva et al. [22] reported on the stoichiometry and stability of complexes formed by Co^2+^, Mn^2+^, and Fe^2+^ with various blood serum proteins, including γ-globulin. The number of metal ions bound to γ-globulin was found to be strongly pH-dependent. As the medium became more alkaline, the number of bound ions increased, varying from 15 to approximately 100 for Co^2+^ (at pH 3.5 and 10.0, respectively), 14 to 23 for Mn^2+^ (at pH 3.0 and 9.5, respectively), and 6 to 27 for Fe^2+^ (at pH 2.6 and 9.1, respectively) [22]. Later research was conducted primarily by Cheknev et al. [23,24,25], who determined that human γ-globulin binds 20 to 30 copper(II) ions [23,24] and 40 to 60 zinc(II) ions [24,25], with equilibrium constants ranging from 3.6 to 6.0 log units (the precise values depending on the experimental technique and stoichiometric model used for data fitting) [24]. The same group also provided a detailed analysis of how interactions with copper(II) or zinc(II) ions alter the immune-related properties of these proteins [26,27,28,29].

Given the functional significance of metal ion interactions with γ-globulin for immune activity, coupled with the growing interest in gold(III)-based therapeutics, this paper investigates the interactions of [AuCl_4_]^−^, [AuCl_3_(OH)]^−^, and [AuCl_2_(OH)_2_]^−^ with γ-globulins from bovine (hereafter BGG) and human (hereafter HGG) blood serum. We determine the stoichiometry of the complexes formed and their apparent binding equilibrium constants.

The focus on γ-globulins from two sources is motivated by the following considerations: first, HGG is the clinically relevant system for understanding potential immunomodulatory effects of gold-based therapeutics in humans. Second, BGG serves as a widely available, structurally homologous model protein with high sequence similarity to HGG in conserved immunoglobulin domains, allowing for comparative binding studies under controlled conditions.

## 2. Results and Discussion

As in a previous study on the interaction of [AuCl_4_]^−^, [AuCl_3_(OH)]^−^, and [AuCl_2_(OH)_2_]^−^ with bovine and human serum albumins [30] (note that the formation of mixed chloride-hydroxide gold(III) complexes other than these is negligible at pH 4) the addition of bovine or human γ-globulin induces a progressive decrease in the characteristic absorbance bands of tetrachloroaurate(III). Specifically, the band at 313 nm (attributed to [AuCl_4_]^−^) and the band at 289 nm (attributed to the mixture of hydrolyzed forms, [AuCl_3_(OH)]^−^ and [AuCl_2_(OH)_2_]^−^) both diminish over time (see Figure S1 for an example). We attribute these spectral changes to the slow, sequential substitution of chloride ligands by N- and O-donor atoms from the protein within the gold(III) coordination sphere [30]. The involvement of other functional groups (e.g., thiols, disulfides) is also plausible and has been reported to lead to the reduction of gold(III) to gold(I) [31,32] or even gold(0). The formation of gold(0) nanoparticles during the kinetic experiments was ruled out, as no absorption band near 550 nm (characteristic of colloidal Au nanoparticles—see, e.g., Figure S4 in Ref. [33]) was observed when the final UV-Vis spectra were recorded over the 200 to 700 nm range. The possibility of reduction to gold(I) will be discussed in greater detail later.

**Figure 1:**
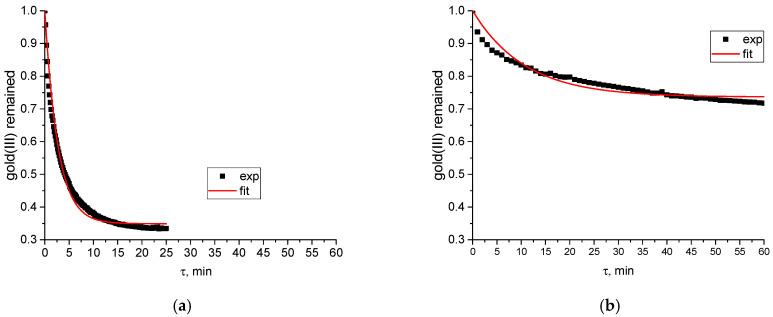
Experimental and fitted kinetic curves characterizing free gold(III) species (2.01 × 10^−4^ mol L^−1^) depletion induced by: (**a**) human serum albumin (3.08 × 10^−5^ mol L^−1^) (data taken from [30]); (**b**) human γ-globulin (3.14 × 10^−5^ mol L^−1^) addition in aqueous solution.

A distinct kinetic difference exists between the binding of gold(III) by albumins and γ-globulins. The reaction of tetrachloroaurate(III) or its hydrolyzed forms (for details on hydrolysis, see Refs. [21,30]) with bovine or human serum γ-globulins proceeds even more slowly than the corresponding reaction with serum albumins [30]. This was quantified by calculating the apparent rate constants for free gold(III) depletion in the presence of human serum albumin (HSA) and γ-globulin (HGG) at roughly equal protein concentrations (3.08 × 10^−5^ mol L^−1^ HSA and 3.14 × 10^−5^ mol L^−1^ HGG). The efficient rate constant for HGG was approximately ten times lower than that for HSA (0.025 min^−1^ vs. 0.246 min^−1^; see Figure 1 for experimental and fitted kinetic curves).

The experimental data for HSA shown in Figure 1 were taken from our previous study [30]. Kinetic data were processed using a simple model of a single reversible gold(III) depletion reaction.

The lower conditional rate constant for tetrachloroaurate(III) binding to γ-globulins, compared to albumin, suggests that under kinetic control, gold(III) complexes would preferentially associate with albumins. It is important to note, however, that following administration, gold(I) species can circulate in the bloodstream for days or even weeks. The half-life of gold-containing drugs in serum is ligand-dependent, as evidenced by a comparison of reported values for auranofin (16 to 25 days [34]; more recent estimates range from 11 to 33 days [35]), aurothiomalate (5.5 days [34]) and other injectable gold salts such as aurothiosulfate and aurothioprol (5 to 7 days [35]). Pharmacokinetic data for gold(III) complexes are scarce, though the available literature [36] indicates that the elimination rates of gold(I) and gold(III) compounds from the body (at least, in mice) are not significantly different. Consequently, the distribution of protein-bound gold is likely governed by differences in binding affinity among serum proteins as well as their concentrations, rather than by kinetics, underscoring the need to determine equilibrium constants (apparent, at least).

**Figure 2:**
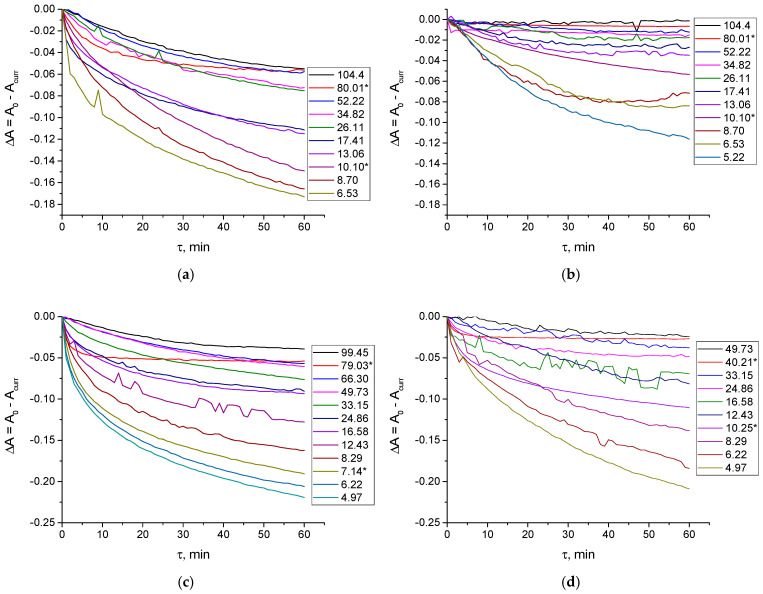
Dependence of difference between starting and current absorbance at λ = 289 nm (**a**,**c**), 313 nm (**b**,**d**) on time of 2.01 × 10^−4^ mol L^−1^ tetrachloroaurate(III) solution in pure distilled water (**a**,**c**) and aqueous 0.1 M NaCl solution (**b**,**d**) upon addition of different amounts of bovine (**a**,**b**) and human (**c**,**d**) γ-globulin. Values in the legend show the ratio of C^0^(H[AuCl_4_]) to C^0^(protein). The asterisks denote the concentration ratios for the additional experiments where the protein concentration was fixed at 2.140 × 10^−6^ mol L^−1^ (BGG) or 3.459 × 10^−6^ mol L^−1^ (HGG), while the tetrachloroaurate(III) concentration varied from 2.161 × 10^−5^ to 2.734 × 10^−4^ mol L^−1^.

Aside from the slower reaction rates, no significant differences were observed in the reactions of gold(III) with the different proteins. Therefore, we applied the same approach as in our previous work [30]. Specifically, a fixed concentration of H[AuCl_4_] (2.01 × 10^−4^ mol L^−1^) in either diluted or 0.1 M NaCl aqueous solution was mixed with increasing concentrations of BGG (0.19 × 10^−5^ to 3.71 × 10^−5^ mol L^−1^) or HGG (0.20 × 10^−5^ to 3.90 × 10^−5^ mol L^−1^). Spectrophotometric kinetic curves were recorded over 1 h (see example data in Figure 2).

**Figure 3:**
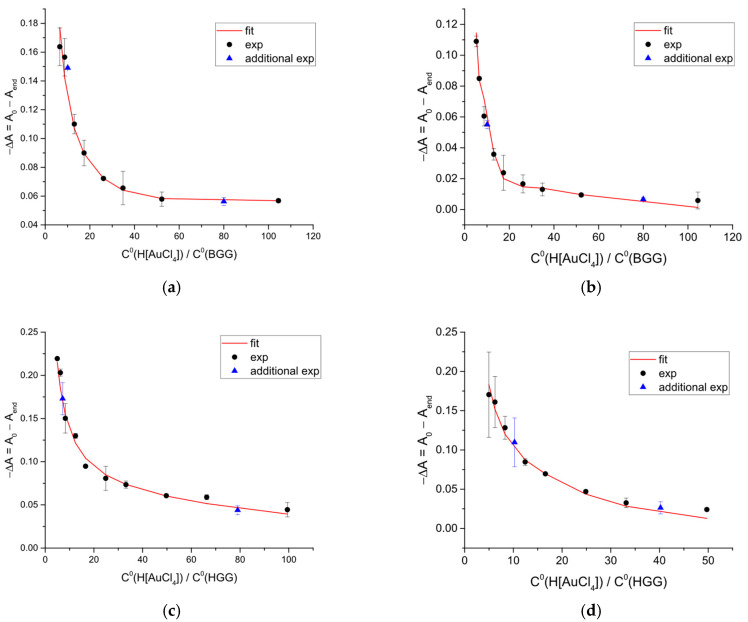
Dependence of the difference between the starting and final absorbance value at λ = 289 nm (**a**,**c**) and 313 nm (**b**,**d**) on ratio of gold(III) to BGG (**a**,**b**) or HGG (**c**,**d**) total concentration (points) in pure distilled water (**a**,**c**) or aqueous 0.1 M NaCl solution (**b**,**d**). Red line shows best fit corresponding to the calculated equilibrium binding constants of gold(III) species to proteins. Error bars are the standard deviations calculated for triplicated experiments.

The difference between the initial and final absorbance values was then plotted against the molar ratio of total gold(III) to protein (Figure 3).

The inflection point visible in Figure 3 indicates the most probable stoichiometric composition of the products formed in the reaction between gold(III) complexes and γ-globulins, specifically the number of gold(III) ions bound per protein molecule. In particular, the points comprising two different branches of the curves in Figure 3 were subjected to linear fitting. The intersections point of these two straight lines allowed for identifying the precise position of the inflection point, while the errors of linear regression parameters contributed to the standard deviation of the final stoichiometry of the products. Based on this analysis, 13 to 19 gold atoms can be bound per γ-globulin molecule.

Previous studies on gold(III) binding to human serum albumin (HSA) have proposed a mechanism involving reduction. Mironov et al. [31,32] suggested that the free thiol group of HSA first reduces gold(III) to gold(I) while being oxidized to a sulfoxide. Because the sulfoxide has low affinity for gold(I), a second albumin molecule is required to stabilize the gold(I) cation via binding it into the stable complex through intact thiol group. This mechanism would imply a stoichiometry of one gold atom per two protein molecules, which is inconsistent with the inflection point observed here or in our previous work on albumin [30]. In contrast, studies on bovine serum albumin (BSA) have shown that, while reduction to gold(0) can occur under alkaline conditions [37], Au(III) species can bind to multiple protein sites without reduction. These sites include aspartate residues, Cys34, and solvent-accessible disulfide bonds [37]. Estimations of gold(III) binding capacity for albumin (less than 30 ions per molecule [37]) are in agreement with our results (5 to 20 ions per molecule [30]). As noted by the authors of that study [37], a crystallographic investigation of the gold(III)-albumin adduct is needed to definitively identify the binding sites.

While the preceding discussion pertains primarily to albumin, similar considerations likely apply to γ-globulins. It has been reported that IgG contains fewer free thiol groups than albumin, approximately 0.24 per protein molecule, as determined after 30 min of incubation with DTNB (5,5′-dithio-bis(2-nitrobenzoic acid)) [38]. Prolonged exposure (24 h) to DTNB yielded about 1.5 thiolate anions per IgG molecule, indicative of disulfide bond cleavage [38]. In any case, a reductive mechanism is unlikely to account for the inflection points observed in the plot of absorbance change versus the gold(III)-to-γ-globulin ratio. This is because even accounting for potential disulfide reduction, the number of available thiol groups remains insufficient. Therefore, we attribute the observed time-dependent changes in the UV-Vis spectra of gold(III) species primarily to the slow substitution of chloride ligands by N- and O-donor atoms from the protein. Nevertheless, the further EXAFS/XANES experiments seem to be crucial in resolving the oxidation state and coordination surrounding of gold ions within the protein solution.

Using the absorbance changes from the kinetic experiments (Figure 3), the total concentrations of H[AuCl_4_] and protein, and the stoichiometry derived from Figure 3, we calculated the equilibrium constants for the formation of gold(III)-protein adducts (Table 1) with the KEV software [39]. The hydrolysis of tetrachloroaurate(III) to [AuCl_3_(OH)]^−^ and [AuCl_2_(OH)_2_]^−^ was accounted for as described previously [30].

The equilibrium constants calculated from spectrophotometric kinetic data are in satisfactory agreement (within the range of standard deviations in most cases) with those determined independently from fluorescence and CD (circular dichroism) spectra. These latter spectra were recorded for a series of solutions containing H[AuCl_4_] and γ-globulins, with the molar ratio of total gold(III) to protein varying from 0 to 32 (see examples of primary data in Figures S2 and S3). Furthermore, data points from control experiments using a fixed protein concentration and varying tetrachloroaurate(III) concentration align with the trend established by the main experiments, providing additional confirmation of experimental consistency.

The equilibrium constants decrease slightly in the order [AuCl_4_]^−^ > [AuCl_3_(OH)]^−^ > [AuCl_2_(OH)_2_]^−^. This trend reflects the higher stability of the hydroxylated complexes relative to the chloride complex, indicating that hydroxide ions are more difficult to displace from the gold(III) coordination sphere than chloride ions [21].

A comparison with our previous results for serum albumins [30] shows that the equilibrium constants and stoichiometries for gold(III)-protein adducts are roughly similar for both albumin and γ-globulin. However, because albumin is present at a higher concentration in blood serum and the kinetics of gold(III) binding favor albumin, gold(III) ions are expected to distribute preferentially to albumin. This conclusion is consistent with an earlier study [40], which examined blood serum from healthy donors after in vitro incubation with various gold-based drugs. Specifically, gold derived from H[AuCl_4_] was found to bind predominantly to albumin (94%), with only a minor fraction associating with globulins (6%) [40].

It should be noted, however, that the distribution of protein-bound gold varies significantly with the drug administered. For the gold(I) drug aurothiomalate, approximately 40% of the protein-bound gold was associated with albumin after 24 h incubation with whole blood, about 29% with α_1_-globulin, and only 6.1% with γ-globulin [34]. In contrast, for auranofin, approximately 84% of the protein-bound gold was attached to globulins, with 51% specifically in the β-globulin fraction. The remainder was nearly equally distributed among albumin, α_2_-globulin, and γ-globulin, showing the least affinity for α_1_-globulin [34].

However, based on the evidence presented, tetrachloroaurate(III) and its hydrolyzed forms, [AuCl_3_(OH)]^−^ and [AuCl_2_(OH)_2_]^−^, are unlikely to react strongly with γ-globulins. Therefore, gold(III)-based drugs may be considered relatively safe in terms of potential side effects on the immune system.

An additional distinction between albumins and γ-globulins is the relative insensitivity of γ-globulin binding to electrolyte concentration; the stoichiometry of the associates formed is approximately the same in water and in 0.1 M NaCl aqueous solution.

Our stoichiometric data (Table 1) are consistent with the findings of Cheknev et al., who reported that γ-globulin binds 20 to 30 copper(II) ions [23,24], and 40 to 60 zinc(II) ions [24,25]. Less number of more bulky complex gold(III) ions bound to proteins is expected.

Beyond providing independent confirmation of the equilibrium constant values, processing of the fluorescence and CD spectral data using the KEV software [39] allowed the determination of the spectra corresponding to hypothetical 1 M solutions of the free protein and its gold(III) complex (Figures S4 and S5). As seen in Figures S2 and S4, gold(III) binding quenches the intrinsic fluorescence of γ-globulins. This quenching is likely a consequence of conformational changes in the protein, suggesting a static quenching mechanism [41,42]; however, a dynamic component cannot be ruled out without fluorescence lifetime measurements [42]. A similar static quenching effect was observed for a series of platinum(II) and palladium(II) complexes that are isostructural and isoelectronic [43] to gold(III) species studied by us. The hypothesis that quenching results from a conformational change altering the fluorophore microenvironment can be further tested using CD spectroscopy, as the spectra (Figure S5) allow for evaluation of gold(III)-induced changes in protein secondary structure.

For this purpose, the secondary structure was analyzed using the BeStSel software [44,45,46]. Our results for free γ-globulins are in agreement with the literature. IgG, the predominant protein in the γ-globulin fraction, is known to consist largely of β-sheets, with reported contributions of approximately 70% [47,48,49,50,51]. The experimental CD spectra recorded in this study (Figure S3) are consistent with published spectra for γ-globulins (see, e.g., Figure 9b in Ref. [52]) and IgG (see, e.g., Figure 2b in Ref. [53] and Figure 3 in Ref. [54]). Characteristic features include a negative peak near 217 nm and a positive band around 200 nm. The intensity of the negative peak diminished progressively with increasing tetrachloroaurate(III) concentration. A similar decrease was reported upon the addition of nanoparticles [53], whereas quantum dots induced negligible changes in the CD spectra of γ-globulin solutions [52].

Analysis with BeStSel indicates that gold(III) addition induces moderate changes in the secondary structure of γ-globulins (Table 2). Specifically, the β-sheet content decreases, while the proportion of disordered fragments (in the absence of NaCl) or turns (in 0.1 M NaCl solution) increases. The transfer of the protein from a dilute solution to 0.1 M NaCl medium does not significantly alter its secondary structure.

It is important to note that CD spectroscopy provides an estimate, rather than a precise determination, of protein secondary structure. The results are sensitive to factors such as the selected wavelength range, spectral intensity, and noise [55]. This consideration is particularly relevant here, as the Author of Ref. [55] also employed the BeStSel algorithm for their analysis. The reported uncertainty for individual structural elements can be as high as 15% [56].

It is noteworthy that the observed changes in the secondary structure of proteins induced by gold addition, though small, can affect their biological functions. The decrease in β-sheet content with the increase in disordered fragments caused by influence of gold(III) species is similar to the thermal denaturation [49] and might be instrumental as the Fab and Fc regions in antibodies are composed mostly of β-sheets [57]. 

**Shortcomings and limitations:** It is important to emphasize that the experimental conditions used in this study to investigate the interactions of tetrachloroaurate(III) and its hydrolyzed species with serum γ-globulins differ significantly from physiological conditions. First, the experiments were conducted at 25 °C, which is lower than the human (and many mammalian) physiological temperature of 37 °C. This difference strongly influences the kinetic data; the reactions studied here would proceed considerably faster in vivo. Second, the pH was maintained at approximately 4 to suppress tetrachloroaurate(III) hydrolysis, whereas the physiological pH is around 7.4. At neutral pH, γ-globulins are nearly uncharged, as their isoelectric point (~7.8) [58] is close to physiological pH. In the acidic medium used, however, the proteins likely carry a net positive charge, which could enhance binding to the anionic gold(III) complexes. Consequently, the apparent equilibrium constants determined here may be higher than those under physiological conditions. Finally, real blood serum contains a multitude of other compounds, both low- and high-molecular-weight, which could compete with or otherwise interfere with the interaction between gold(III) species and the target proteins.

## 3. Materials and Methods

### 3.1. Materials

Hydrogen tetrachloroaurate(III) trihydrate (H[AuCl_4_]·3H_2_O; LenReaktiv, Saint-Petersburg, Russia; claimed Au content: 49.11%), human serum γ-globulin (HGG; Sigma, St. Louis, MO, USA; purity ≥ 96%), and bovine serum γ-globulin (BGG; Maklin, Shanghai, China; purity ≥ 96%) were used as received. Sodium chloride (NaCl; Labtekh, Moscow, Russia) was recrystallized from deionized water prior to use. All solutions for spectroscopic studies were prepared using bidistilled water (specific conductivity, *κ* = 3.6 μs cm^−1^; pH 6.6). Working protein solutions were prepared with the addition of sodium perchlorate (NaClO_4_) to a final concentration of ~0.01 mol L^−1^ to ensure solubility (γ-globulins are poorly soluble in distilled water without electrolytes added) and were stored at 4 °C. Protein concentrations were determined spectrophotometrically immediately before each experiment, using a molar extinction coefficient (*ε*_280_) of 210 000 L mol^−1^ cm^−1^ [59].

### 3.2. Methods

All UV-Vis experiments were performed using a double-beam Shimadzu UV1800 spectrophotometer (Shimadzu, Marlborough, MA, USA). Measurements were conducted in the wavelength range of 200–500 nm with an absorbance range of 0–1.3. For kinetic studies, varying amounts of HGG (0.20 × 10^−5^ to 3.90 × 10^−5^ mol L^−1^) or BGG (0.19 × 10^−5^ to 3.71 × 10^−5^ mol L^−1^) were added to an aqueous solution of H[AuCl_4_] (2.01 × 10^−4^ mol L^−1^). The absorbance of H[AuCl_4_] was monitored at *λ* = 289 nm (in diluted solution) or *λ* = 315 nm (in 0.1 M NaCl aqueous solution) for a duration of 3600 s with a sampling interval of 60 s. The wavelength determination error did not exceed ±0.5 nm, and the maximum absorbance measurement inaccuracy was ±0.003 units. The temperature was maintained at 298.2 ± 0.1 K using an external thermostat. Quartz cuvettes with a 1.00 cm optical path length were used. All experiments were performed in duplicate at minimum. Kinetic data were processed using Kinet software, version 0.8 [60], to calculate the rate constants.

Fluorescence spectra were recorded using a CM2203 spectrofluorometer (Solar, Minsk, Belarus) for aqueous solutions containing HGG or BGG (1.24 to 1.27 × 10^−5^ mol L^−1^) and varying concentrations of H[AuCl_4_] (0.794, 1.580, 2.350, 3.11, and 3.870 × 10^−4^ mol L^−1^), either in diluted aqueous solution or in 0.1 M NaCl medium. The excitation wavelength was set at 280 nm, and emission was monitored from 290 to 400 nm. All solutions were equilibrated for 2 h prior to measurement. The excitation and emission slit widths were set at 5 nm, and quartz cuvettes with a 1.00 cm optical path length were used. All experiments were performed in triplicate.

The inner filter effect due to increasing tetrachloroaurate(III) concentration was corrected using the following equation [41,42]:

(1) $$F_{corr}=F_{obs}\cdot 10^{0.5(A_{ex}+A_{em})}$$

where *F_corr_* and *F_obs_* are the corrected and observed fluorescence intensities, respectively, and *A_ex_* and *A_em_* are the absorbance values at the excitation and emission wavelengths, respectively.

Circular dichroism spectra were recorded using a J-1500 CD spectrometer (JASCO, Portland, OR, USA) under a nitrogen atmosphere. Measurements were performed on aqueous solutions containing BGG (4.28 × 10^−7^ mol L^−1^) or HGG (6.92 × 10^−7^ mol L^−1^) and increasing concentrations of H[AuCl_4_]. For BGG experiments, H[AuCl_4_] concentrations were 0.28, 0.56, 0.83, 1.11, and 1.39 × 10^−5^ mol L^−1^. For HGG experiments, the corresponding concentrations were 0.43, 0.87, 1.30, 1.73, and 2.16 × 10^−5^ mol L^−1^. Measurements were carried out either in pure water or in 0.1 M NaCl medium, in the wavelength range of 170–250 nm. Quartz cuvettes with a 1.00 cm optical path length were used, and all experiments were conducted at least in duplicate.

In all spectral experiments, the pH was approximately 4 due to the acidic dissociation of tetrachloroaurate(III). This pH effectively suppresses the hydrolysis of [AuCl_4_]^−^ at pCl = 1, as the hydrolysis equilibrium depends on the sum pH + pCl [61]. Solution acidity was monitored potentiometrically.

## 4. Conclusions

UV-Vis spectroscopy revealed slow reactions between gold(III) species such as tetrachloroaurate(III) in 0.1 M NaCl and its hydrolyzed forms, [AuCl_3_(OH)]^−^ and [AuCl_2_(OH)_2_]^−^, in diluted aqueous solution, and bovine or human serum γ-globulins. Analysis of the absorbance change as a function of the gold(III)-to-protein molar ratio determined the binding stoichiometry to be 13.3 ± 3.3 to 18.9 ± 5.1 gold(III) ions per protein molecule. This stoichiometry was independent of the protein source (bovine/human) and the medium (low electrolyte concentration or 0.1 M NaCl solution). The conditional equilibrium constants per gold(III) ion followed the order [AuCl_4_]^−^ > [AuCl_3_(OH)]^−^ > [AuCl_2_(OH)_2_]^−^, with log *K*’ values ranging from 4.6 (for BGG with [AuCl_4_]^-^) down to 3.6 (for HGG with [AuCl_2_(OH)_2_]^−^). This trend reflects the higher thermodynamic stability of the hydroxylated complexes, making hydroxide ligands more difficult to displace than chlorides.

Gold(III) binding quenches the intrinsic fluorescence of both γ-globulins and induces moderate changes in their secondary structure, specifically a decrease in β-sheet content and a concurrent increase in turns or disordered fragments. The transfer of proteins from dilute solution to 0.1 M NaCl medium had a negligible effect on their secondary structure.

It is important to note that these results were obtained under non-physiological conditions and warrant careful interpretation. In vivo, factors such as higher temperature (37 °C) could accelerate binding kinetics, while the neutral physiological pH (reducing the net positive charge on the protein) and the presence of competing serum components could diminish the apparent affinity. Despite these differences, the findings are consistent with the known pharmacokinetics and serum biodistribution of gold-based drugs, which show preferential distribution to other serum proteins, particularly albumin.

## Figures and Tables

**Table 1:** Stoichiometry and apparent stability constants of the complexes formed by [AuCl_4_]^−^, [AuCl_3_(OH)]^−^ and [AuCl_2_(OH)_2_]^−^ with bovine and human γ-globulin at *T* = 298.2 K, *p* = 0.1 MPa, pH ~4.

Medium	Gold(III) Species	Gold(III) Ions per 1 Protein Molecule *	log *K*’ per 1 Gold(III) Ion			Gold(III) Ions per 1 Protein Molecule *	log *K*’ per 1 Gold(III) Ion		
			UV-Vis	Fluorimetry	CD		UV-Vis	Fluorimetry	CD
		Bovine γ-Globulin				Human γ-Globulin			
0.1 M NaCl	[AuCl_4_]^−^	15.2 ± 5.1	4.64 ± 0.01	4.40 ± 0.11	4.46 ± 0.09	13.3 ± 3.3	4.18 ± 0.28	3.71 ± 0.02	4.07 ± 0.09
No NaCl added	[AuCl_3_(OH)]^−^	11.3 ± 3.4	4.09 ± 0.12	4.21 ± 0.09	4.07 ± 0.08	10.3 ± 1.2	3.85 ± 0.24	4.27 ± 0.09	4.02 ± 0.29
	[AuCl_2_(OH)_2_]^−^	7.6 ± 1.7	3.89 ± 0.12	4.02 ± 0.09	3.89 ± 0.08	6.8 ± 0.6	3.63 ± 0.24	4.08 ± 0.09	3.81 ± 0.27

* the values are calculated using the position of the inflection points in Figure 3.

**Table 2:** Secondary structure of bovine and human γ-globulin as well as their association products with gold(III) species in water and aqueous 0.1 M NaCl determined from CD spectra.

Secondary Structure Element	BGG in Diluted Solution	BGG + Gold(III) in Diluted Solution	BGG in 0.1 M NaCl	BGG + Gold(III) in 0.1 M NaCl	HGG in Diluted Solution	HGG + Gold(III) in Diluted Solution	HGG in 0.1 M NaCl	HGG + Gold(III) in 0.1 M NaCl
α-helices, %	0	1.3	0	0	0	8.5	0	0
β-sheets, %	64.9	43.9	55.6	48.6	58.7	41.3	58.6	47.4
turns, %	15.8	16.2	8.8	14.2	7.1	7.1	6.3	18.6
other, %	19.2	38.6	35.6	37.2	34.2	43.1	35.0	34.0

## Data Availability

Data is contained within the article or Supplementary Materials. Further inquiries can be directed to the corresponding author.

## Supplementary Materials

The following supporting information can be downloaded at: https://www.mdpi.com/article/10.3390/ijms27010541/s1.

## Abbreviations

The following abbreviations are used in this manuscript:

BGG	Bovine γ-globulin
HGG	Human γ-globulin
CD	Circular dichroism
BSA	Bovine serum albumin
HSA	Human serum albumin

## References

[1] Varga M. (2014). Clinical Pathology. Textbook of Rabbit Medicine.

[2] Najjar V.A., Meister A. (1974). The Physiological Role of γ-Globulin. Advances in Enzymology- and Related Areas of Molecular Biology.

[3] Glover Z.K., Basa L., Moore B., Laurence J.S., Sreedhara A. (2015). Metal Ion Interactions with mAbs: Part 1: pH and Conformation Modulate Copper-Mediated Site-Specific Fragmentation of the IgG1 Hinge Region. mAbs.

[4] Gupta S., Upadhyay K., Schöneich C., Rathore A.S. (2022). Impact of Various Factors on the Kinetics of Non-Enzymatic Fragmentation of a Monoclonal Antibody. Eur. J. Pharm. Biopharm..

[5] Hou W., Wu S., Liu Y., Li H. (2022). Impact of Conformational Change of Immunoglobulin G Induced by Silver Ions on Escherichia Coli and Macrophage Adhesion to Biomaterial Surfaces. Colloids Surf. A Physicochem. Eng. Asp..

[6] Saporito-Magriña C., Facio M.L., Lopez-Montañana L., Pagano G., Repetto M.G. (2023). Copper-Induced Aggregation of IgG: A Potential Driving Force for the Formation of Circulating Protein Aggregates. Metallomics.

[7] Messori L., Marcon G., Orioli P. (2003). Gold(III) Compounds as New Family of Anticancer Drugs. Bioinorg. Chem. Appl..

[8] Gabbiani C., Casini A., Messori L. (2007). Gold(III) Compounds as Anticancer Drugs. Gold Bull.

[9] Zhou X.-Q., Abyar S., Carbo-Bague I., Wang L., Türck S., Siegler M.A., Basu U., Ott I., Liu R., IJzerman A.P. (2024). Multitarget Thiol-Activated Tetrapyridyl Gold(III) Complexes for Hypoxic Cancer Therapy. CCS Chem..

[10] Zhang J., Li Y., Fang R., Wei W., Wang Y., Jin J., Yang F., Chen J. (2022). Organometallic Gold(I) and Gold(III) Complexes for Lung Cancer Treatment. Front. Pharmacol..

[11] Gurba A., Taciak P., Sacharczuk M., Młynarczuk-Biały I., Bujalska-Zadrożny M., Fichna J. (2022). Gold (III) Derivatives in Colon Cancer Treatment. Int. J. Mol. Sci..

[12] Ratia C., Sueiro S., Soengas R.G., Iglesias M.J., López-Ortiz F., Soto S.M. (2022). Gold(III) Complexes Activity against Multidrug-Resistant Bacteria of Veterinary Significance. Antibiotics.

[13] Ratia C., Ballén V., Gabasa Y., Soengas R.G., Velasco-de Andrés M., Iglesias M.J., Cheng Q., Lozano F., Arnér E.S.J., López-Ortiz F. (2023). Novel Gold(III)-Dithiocarbamate Complex Targeting Bacterial Thioredoxin Reductase: Antimicrobial Activity, Synergy, Toxicity, and Mechanistic Insights. Front. Microbiol..

[14] Büssing R., Karge B., Lippmann P., Jones P.G., Brönstrup M., Ott I. (2021). Gold(I) and Gold(III) N-Heterocyclic Carbene Complexes as Antibacterial Agents and Inhibitors of Bacterial Thioredoxin Reductase. ChemMedChem.

[15] Yeo C.I., Goh C.H.P., Tiekink E.R.T., Chew J. (2024). Antibiotics: A “GOLDen” Promise?. Coord. Chem. Rev..

[16] Myagkova I.N., Evseev A.K., Polyakov N.A., Drovosekov A.B., Goroncharovskaya I.V., Shabanov A.K. (2022). Physico-chemical approaches to improve the characteristics of electrosurgical instruments. Izv. Vyssh. Uchebn. Zaved. Khim. Khim. Tekhnol..

[17] Wróblewska A., Sadowski M., Jasiński R. (2024). Selectivity and molecular mechanism of the Au(III)-catalyzed [3+2] cycloaddition reaction between (Z)-C,N-diphenylnitrone and nitroethene in the light of the molecular electron density theory computational study. Chem. Heter. Comp..

[18] Shaw C.F. (1999). Gold-Based Therapeutic Agents. Chem. Rev..

[19] Gamov G.A. (2021). Processing of the Spectrofluorimetric Data Using the Graphical Methods and the Maximum Likelihood Approach. Spectrochim. Acta Part A Mol. Biomol. Spectrosc..

[20] Yarullin D.N., Zavalishin M.N., Sharnin V.A., Gamov G.A. (2022). Equilibrium in a Bovine Serum Albumin–Pyridoxal-5-Phosphate 4-Hydroxybenzoyl Hydrazone–La3+ Ion System. Russ. J. Phys. Chem. A.

[21] Gamov G.A. (2024). Complexation of Gold(I) and Gold(III) in Solutions. Coord. Chem. Rev..

[22] Bondareva A.P., Kolosov I.V., Landau M.A. (1980). Complexation of Co(II), Fe(II), and Mn(II) with Serum Proteins. Koord. Khim..

[23] Babaeva E.E., Vorobyova U.A., Zharkova M.S., Cheknyov S.B. (2006). Human Serum γ-Globulin Binds Copper Cations. Bull Exp. Biol. Med..

[24] Cheknev S.B., Babaeva E.E., Golub A.E., Denisova E.A., Vorobieva U.A. (2014). The effects of copper and zinc ions during their binding with human serum γ-globulin. Med. Immunol..

[25] Babaeva E.E., Vorobyova U.A., Denisova E.A., Medvedeva D.A., Cheknev S.B. (2006). Binding of Zinc Cations to Human Serum γ-Globulin. Bull Exp. Biol. Med..

[26] Cheknev S.B., Sarycheva M.A., Mezdrokina A.S., Babayants A.A. (2016). The G-globulin metal complexes in regulation of the production by human blood cells of monocyte chemoattractant protein MCP-1. Immunologiya.

[27] Cheknev S.B. (2021). The Proteins of γ-Globulin Fraction, That Bind Metal Ions, in Physiological Immune Regulation. Opposite Effects of Copper and Zinc. Immunologiya.

[28] Cheknev S.B. (2021). The Proteins of γ-Globulin Fraction Binding Metal Ions, in Physiological Immune Regulation. Mutual Action of Copper and Zinc. Immunologiya.

[29] Cheknev S.B. (2022). The Proteins of γ-Globulin Fraction, That Bind Metal Ions, in Physiological Immune Regulation. Polarization of the Responses and Rational Limitation of Inflammation. Immunologiya.

[30] Zavalishin M.N., Pimenov O.A., Belov K.V., Khodov I.A., Gamov G.A. (2023). Chemical Equilibria in Aqueous Solutions of H[AuCl4] and Bovine or Human Serum Albumin. J. Mol. Liq..

[31] Mironov I.V., Kharlamova V.Y. (2023). On the Interaction of Gold(III) Complexes with Human Serum Albumin. Russ. J. Inorg. Chem..

[32] Mironov I.V., Kharlamova V.Y., Makotchenko E.V. (2024). Some Remarks on the Biological Application of Gold(III) Complexes. Biometals.

[33] Akulinina A.A., Roshchin I.S., Konstantinov L.E., Yarullin D.N., Zavalishin M.N., Kholodkov I.V., Gamov G.A. (2024). Gold(III)-DNA Interaction in Aqueous Solution. J. Mol. Liq..

[34] Iqbal M.S., Taqi S.G., Arif M., Wasim M., Sher M. (2009). In Vitro Distribution of Gold in Serum Proteins after Incubation of Sodium Aurothiomalate and Auranofin with Human Blood and Its Pharmacological Significance. Biol. Trace Elem. Res..

[35] Balfourier A., Kolosnjaj-Tabi J., Luciani N., Carn F., Gazeau F. (2020). Gold-Based Therapy: From Past to Present. Proc. Natl. Acad. Sci. USA.

[36] Tomasello M.F., Nardon C., Lanza V., Di Natale G., Pettenuzzo N., Salmaso S., Milardi D., Caliceti P., Pappalardo G., Fregona D. (2017). New Comprehensive Studies of a Gold(III) Dithiocarbamate Complex with Proven Anticancer Properties: Aqueous Dissolution with Cyclodextrins, Pharmacokinetics and Upstream Inhibition of the Ubiquitin-Proteasome Pathway. Eur. J. Med. Chem..

[37] Dixon J.M., Egusa S. (2018). Conformational Change-Induced Fluorescence of Bovine Serum Albumin–Gold Complexes. J. Am. Chem. Soc..

[38] Schauenstein E., Sorger S., Reiter M., Dachs F. (1982). Free Thiol Groups and Labile Disulfide Bonds in the IgG Fraction of Human Serum. J. Immunol. Methods.

[39] Meshkov A.N., Gamov G.A. (2019). KEV: A Free Software for Calculating the Equilibrium Composition and Determining the Equilibrium Constants Using UV–Vis and Potentiometric Data. Talanta.

[40] Herrlinger J.D., Weikert W. (1982). Protein binding of gold in serum of patients treated with different gold preparations. Z Rheumatol..

[41] Van De Weert M. (2010). Fluorescence Quenching to Study Protein-Ligand Binding: Common Errors. J. Fluoresc..

[42] Van De Weert M., Stella L. (2011). Fluorescence Quenching and Ligand Binding: A Critical Discussion of a Popular Methodology. J. Mol. Struct..

[43] Sookai S., Munro O.Q. (2023). Complexities of the Interaction of Ni^II^, Pd^II^ and Pt^II^ Pyrrole-Imine Chelates with Human Serum Albumin**. ChemistryEurope.

[44] Micsonai A., Wien F., Kernya L., Lee Y.-H., Goto Y., Réfrégiers M., Kardos J. (2015). Accurate Secondary Structure Prediction and Fold Recognition for Circular Dichroism Spectroscopy. Proc. Natl. Acad. Sci. USA.

[45] Micsonai A., Wien F., Bulyáki É., Kun J., Moussong É., Lee Y.-H., Goto Y., Réfrégiers M., Kardos J. (2018). BeStSel: A Web Server for Accurate Protein Secondary Structure Prediction and Fold Recognition from the Circular Dichroism Spectra. Nucleic Acids Res..

[46] Micsonai A., Moussong É., Wien F., Boros E., Vadászi H., Murvai N., Lee Y.-H., Molnár T., Réfrégiers M., Goto Y. (2022). BeStSel: Webserver for Secondary Structure and Fold Prediction for Protein CD Spectroscopy. Nucleic Acids Res..

[47] Buijs J., Norde W., Lichtenbelt J.W.T. (1996). Changes in the Secondary Structure of Adsorbed IgG and F(Ab‘)_2_ Studied by FTIR Spectroscopy. Langmuir.

[48] Li S.-Q., Bomser J.A., Zhang Q.H. (2005). Effects of Pulsed Electric Fields and Heat Treatment on Stability and Secondary Structure of Bovine Immunoglobulin G. J. Agric. Food Chem..

[49] Vermeer A.W.P., Bremer M.G.E.G., Norde W. (1998). Structural Changes of IgG Induced by Heat Treatment and by Adsorption onto a Hydrophobic Teflon Surface Studied by Circular Dichroism Spectroscopy. Biochim. Biophys. Acta (BBA)-Gen. Subj..

[50] Vermeer A.W.P., Norde W. (2000). The Influence of the Binding of Low Molecular Weight Surfactants on the Thermal Stability and Secondary Structure of IgG. Colloids Surf. A Physicochem. Eng. Asp..

[51] Dutta U., Cohenford M.A., Dain J.A. (2006). Monitoring the Effect of Glucosamine and Glyceraldehyde Glycation on the Secondary Structure of Human Serum Albumin and Immunoglobulin G: An Analysis Based on Circular Dichroism, Thermal Melting Profiles and UV–Fluorescence Spectroscopy. Anal. Chim. Acta.

[52] Ba X.-X., Gao T., Yang M., Jiang P., Jiang F.-L., Liu Y. (2020). Thermodynamics of the Interaction Between Graphene Quantum Dots with Human Serum Albumin and γ-Globulins. J. Solution Chem..

[53] Liu W., Rose J., Plantevin S., Auffan M., Bottero J.-Y., Vidaud C. (2013). Protein Corona Formation for Nanomaterials and Proteins of a Similar Size: Hard or Soft Corona?. Nanoscale.

[54] Deokar V., Sharma A., Mody R., Volety S.M. (2020). Comparison of Strategies in Development and Manufacturing of Low Viscosity, Ultra-High Concentration Formulation for IgG1 Antibody. J. Pharm. Sci..

[55] Jones C. (2024). Impact of Imperfect Data on Protein Secondary Structure Estimates from Far-UV Circular Dichroism Spectra. Anal. Biochem..

[56] Nagy G., Grubmüller H. (2020). How Accurate Is Circular Dichroism-Based Model Validation?. Eur. Biophys. J..

[57] Janeway C.A., Travers P., Walport M., Walport M., Shlomchik M. (2001). The structure of a typical antibody molecule. Immunobiology: The Immune System in Health and Disease.

[58] Mizerska U., Fortuniak W., Pospiech P., Chojnowski J., Slomkowski S. (2015). Gamma Globulins Adsorption on Carbofunctional Polysiloxane Microspheres. J. Inorg. Organomet. Polym..

[59] https://assets.thermofisher.com/TFS-Assets/LSG/Application-Notes/TR0006-Extinction-coefficients.pdf.

[60] Абраменкoв A.B. KINET Прoграмма Для Численнoгo Мoделирoвания Кинетики Слoжных Химических Реакций. https://www.chem.msu.ru/rus/teaching/KINET2012/.

[61] Bjerrum N. (1948). La Stabilité Des Chlorures d’or. Bull. Soc. Chim..

