# Supplementary material for: Binding of Tetrachloroaurate(III) to Bovine or Human γ-Globulins
Source: Int J Mol Sci. 2026 Jan 5;27(1):541. doi: 10.3390/ijms27010541 (PMC12786863; doi:10.3390/ijms27010541)
Supplement: Supplementary file 1 [file ijms-27-00541-s001.zip › ijms-4088802-supplementary.pdf]

Binding of Tetrachloroaurate(III) to Bovine or Human γ-Globulins

Daniil N. Yarullin, Olga I. Logacheva, Maksim N. Zavalishin and George A. Gamov *

Department of General Chemical Technology, Ivanovo State University of Chemistry and Technology, Sheremetevskii Ave. 7, 153000 Ivanovo, Russia; yarullin_dn@isuct.ru (D.N.Y.); o.logachiova@yandex.ru (O.I.L.); zavalishin00@gmail.com (M.N.Z.)

* Correspondence: ggamov@isuct.ru; Tel.: +7-(915)-821-85-62

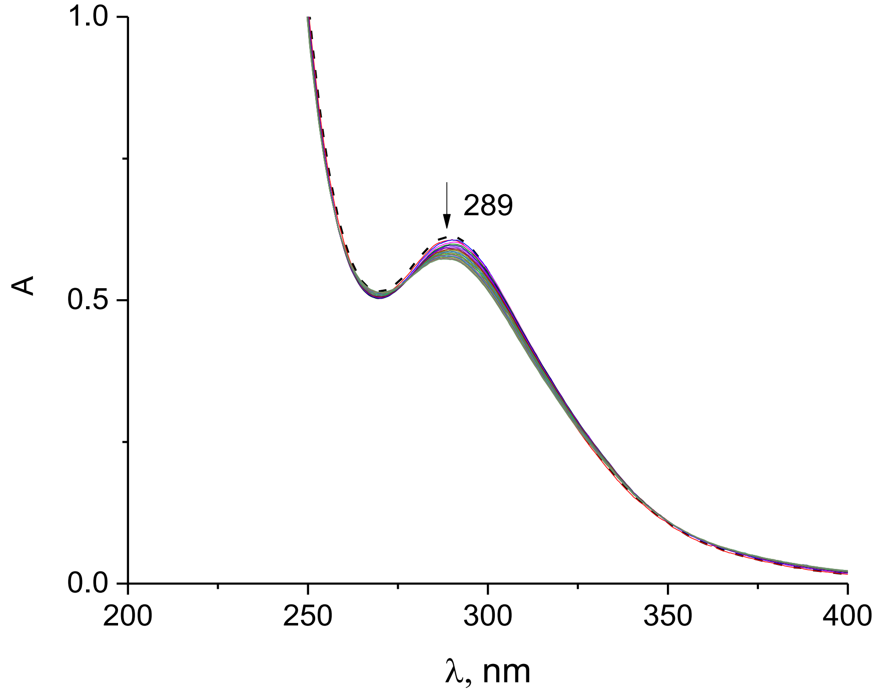

a

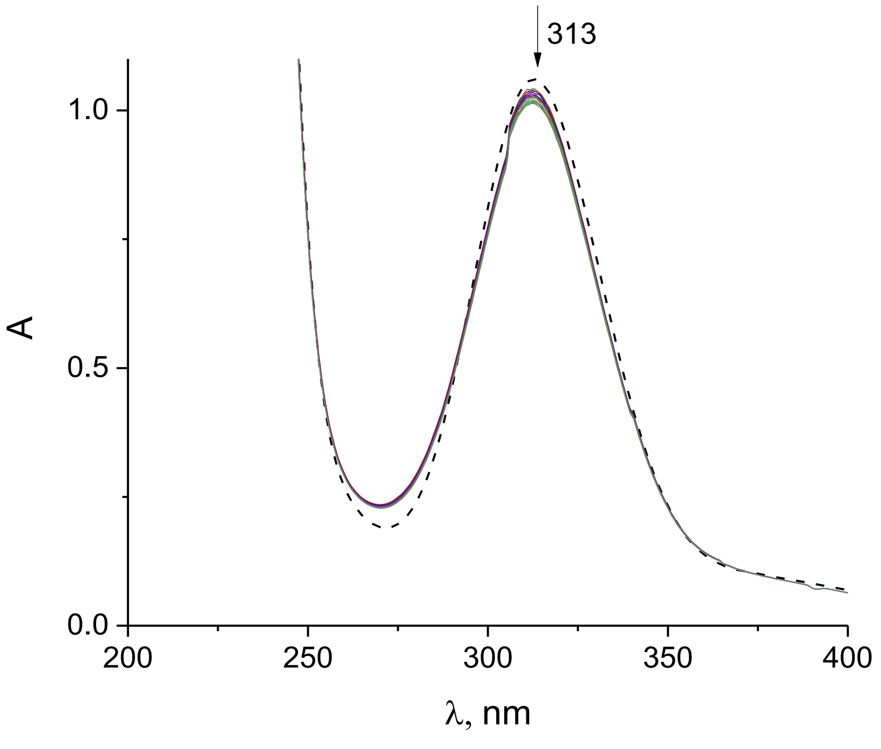

b

Figure S1. The changes in UV-Vis spectra of tetrachloroaurate(III) of 2.01×10^{-4} M induced by addition of 4.0×10^{-6} M of human γ-globulin in: (**a**) pure distilled water; (**b**) in aqueous 0.1 M NaCl solution. Twenty spectra are registered with a delay of 60 s. The spectra of the starting 2.01×10^{-4} M tetrachloroaurate(III) solutions without protein addition are given in bold dashed lines.

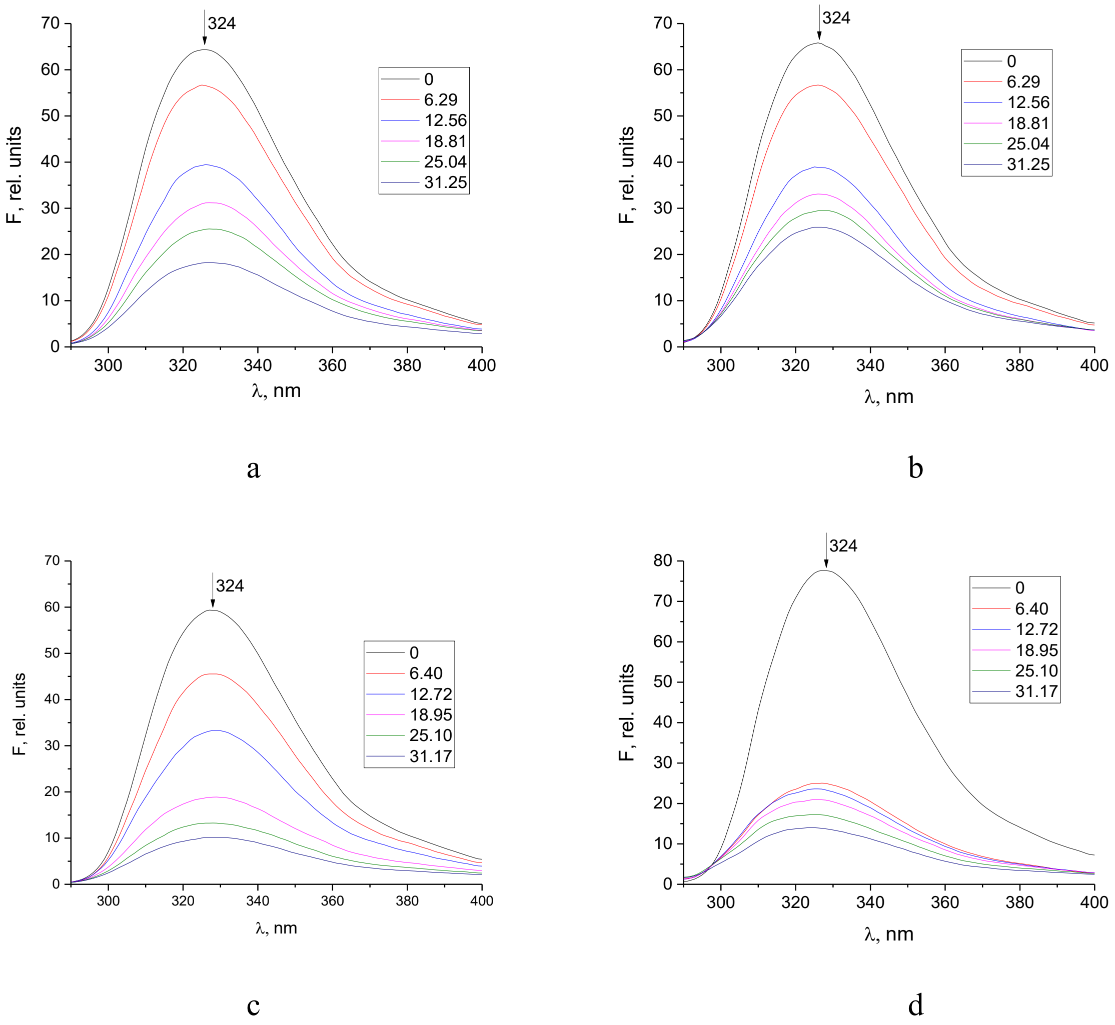

Figure S2. Fluorescent emission spectra of 1.27×10^{-5} M bovine (**a**,**b**) γ-globulin and 1.24×10^{-5} M human (**c**,**d**) γ-globulin solutions in pure distilled water (**a**,**c**) and aqueous 0.1 M NaCl solution (**b**,**d**) upon addition of different amounts of $H[AuCl_4]$. Values in the legend show the ratio of $C^0(H[AuCl_4])$ to C^0(protein).

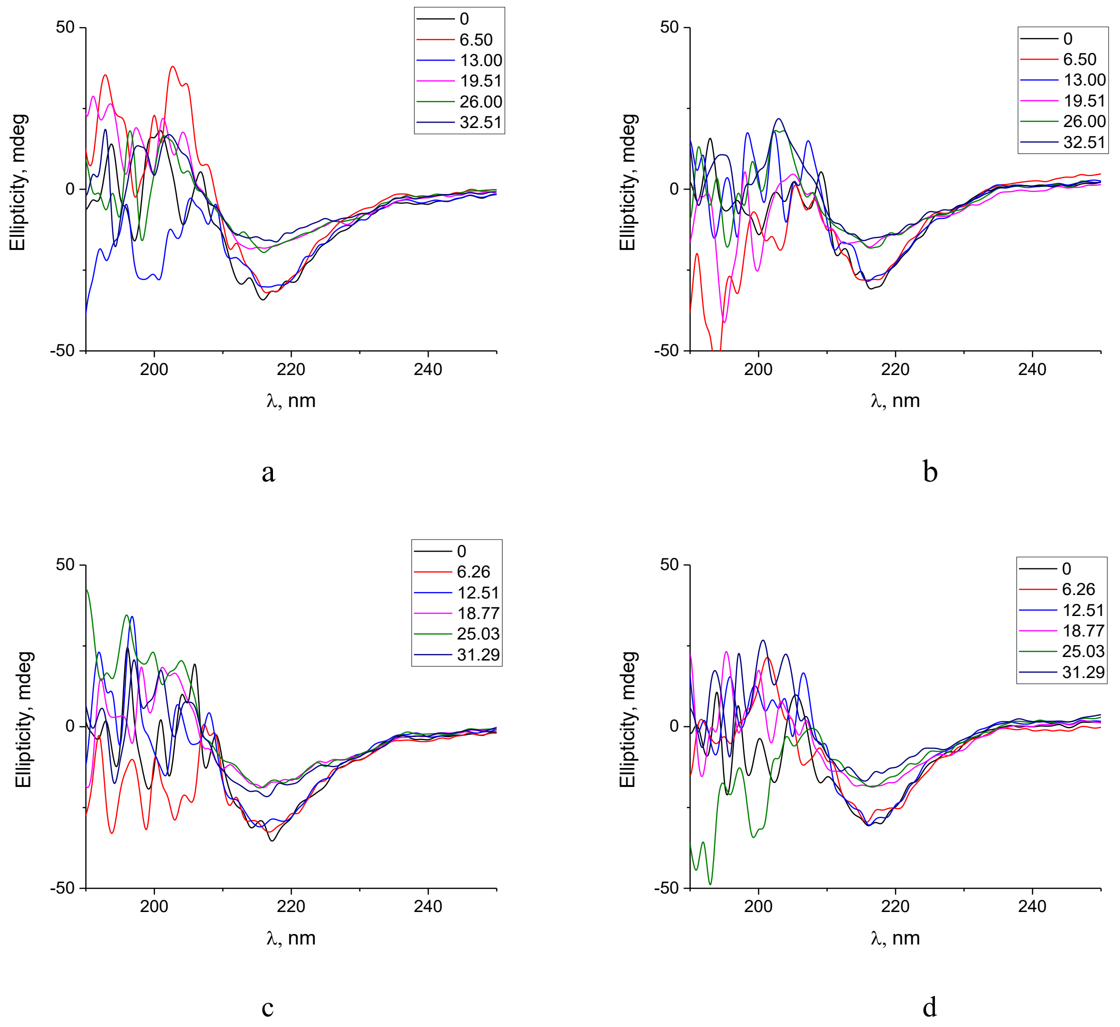

Figure S3. Circular dichroism spectra of 4.28×10^{-7} M bovine (**a**,**b**) γ-globulin and 6.92×10^{-7} M human (**c**,**d**) γ-globulin solutions in pure distilled water (**a**,**c**) and aqueous 0.1 M NaCl solution (**b**,**d**) upon addition of different amounts of $H[AuCl_4]$. Values in the legend show the ratio of $C^0(H[AuCl_4])$ to C^0(protein).

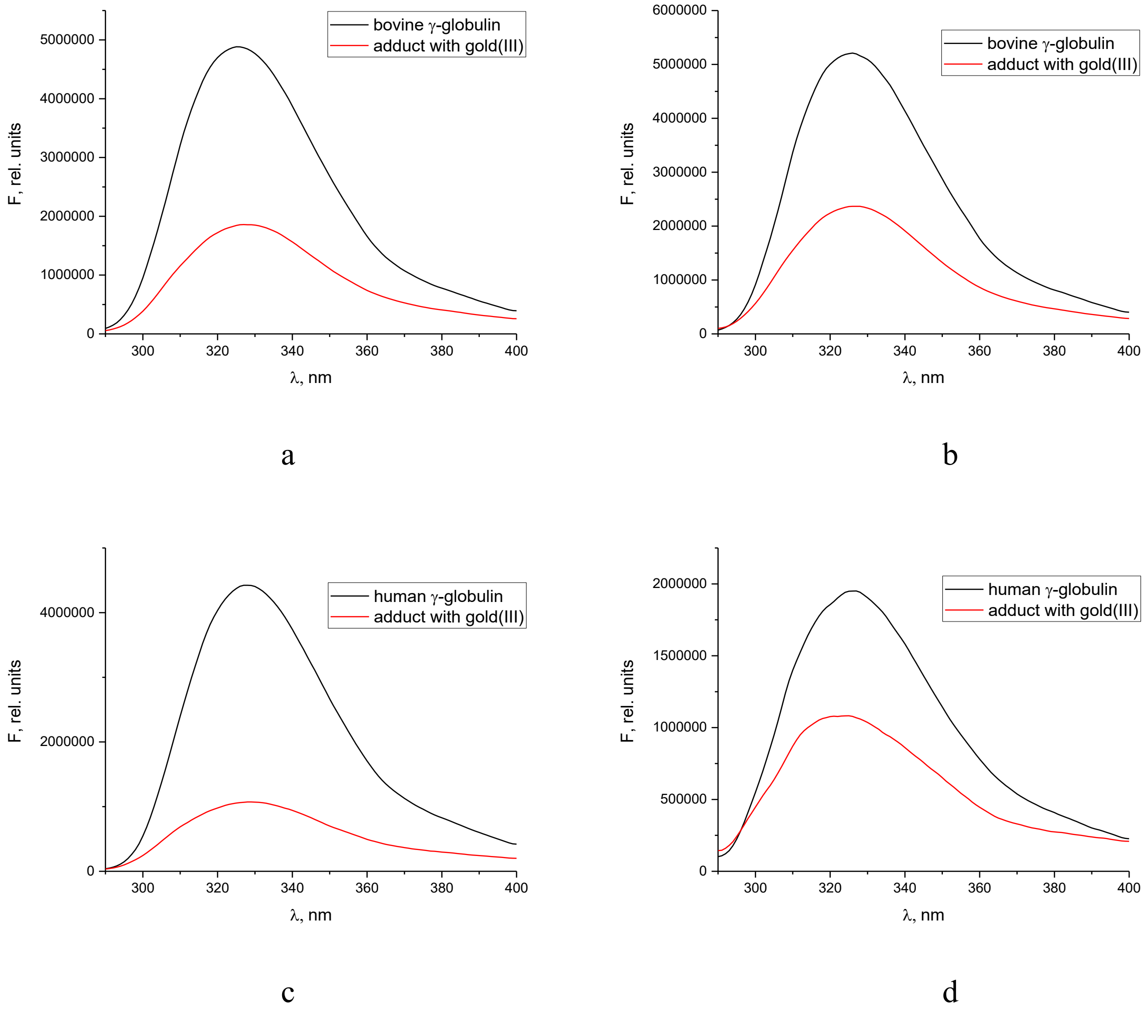

Figure S4. Fluorescent emission spectra of hypothetical 1 M solutions of bovine (**a**,**b**) and human (**c**,**d**) γ-globulin solutions in pure distilled water (**a**,**c**) and aqueous 0.1 M NaCl solution (**b**,**d**) as well as their complexes with gold(III) species.

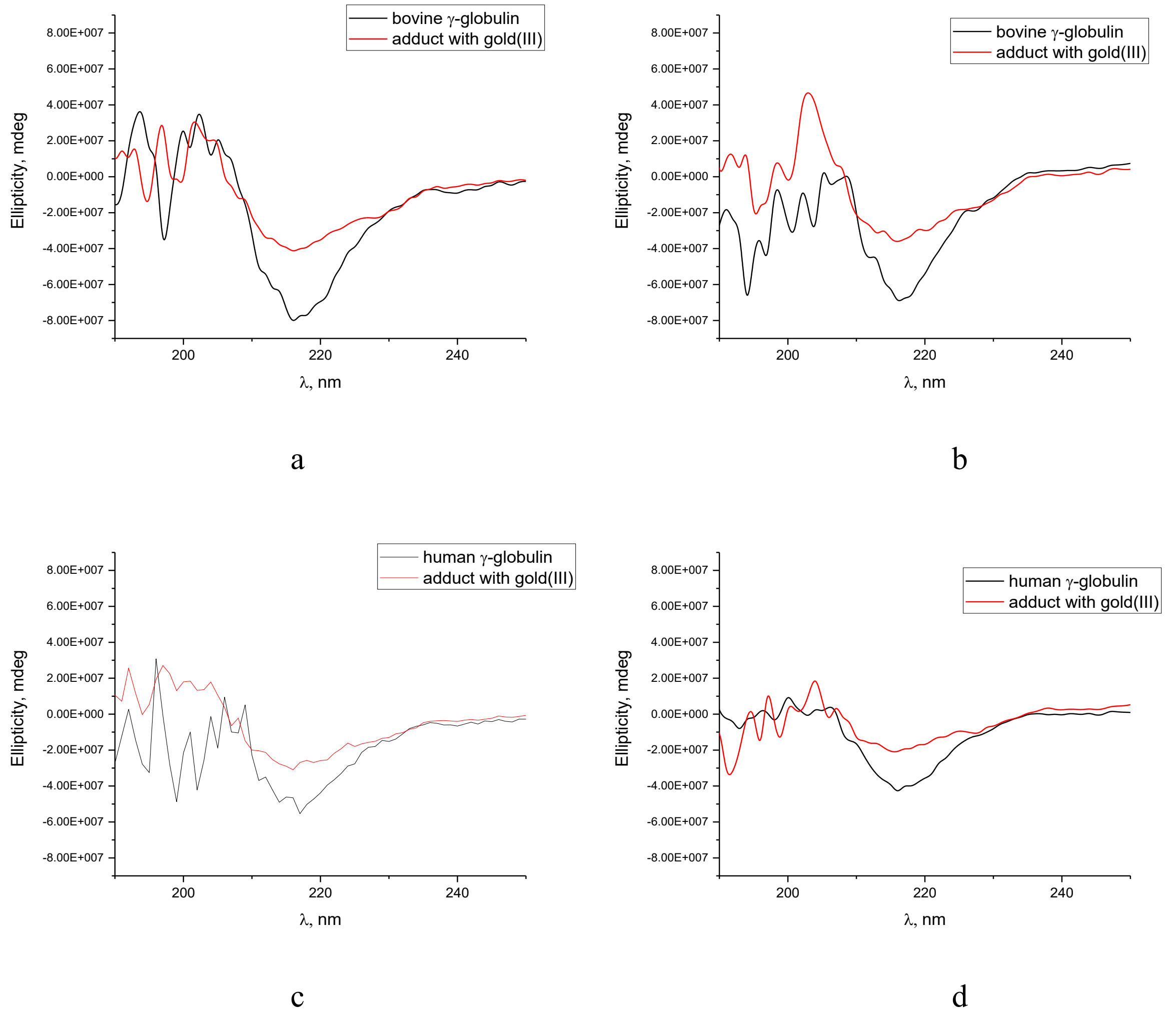

Figure S5. Circular dichroism spectra of hypothetical 1 M solutions of bovine (**a**,**b**) and human (**c**,**d**) γ-globulin solutions in pure distilled water (**a**,**c**) and aqueous 0.1 M NaCl solution (**b**,**d**) as well as their complexes with gold(III) species
